# Latent AKI is… still AKI: the quantification of the burden of renal dysfunction

**DOI:** 10.1186/s13054-016-1428-9

**Published:** 2016-08-26

**Authors:** Zaccaria Ricci, Stefano Romagnoli, Luca Di Chiara

**Affiliations:** 1 Department of Cardiology and Cardiac Surgery, Pediatric Cardiac Intensive Care Unit, Bambino Gesù Children’s Hospital, IRCCS, Piazza S. Onofrio 4, 00165 Rome, Italy; 2 Department of Health Science, University of Florence, Florence, Italy; 3 Department of Anesthesia and Intensive Care, Azienda Ospedaliero-Universitaria Careggi, Florence, Italy

**Keywords:** Acute kidney injury, Congenital heart disease, Cardiopulmonary bypass, Neutrophil gelatinase associated lipocalin

## Abstract

The association between pediatric cardiac surgery, acute kidney injury (AKI), and clinical outcomes has been studied several times in the recent literature. In this issue of *Critical Care* an interesting and original study analyzed the *path* from causal AKI entities to clinical AKI consequences through the application of structural equation modeling. The authors described the complex connections linking duration of cardiopulmonary bypass, cross clamp-time, and descriptors of low cardiac output syndrome to AKI modeled as a complex variable composed of post-operative serum creatinine increase of 50 % over baseline, urine output <0.5 ml/kg/h, and urine creatinine-normalized neutrophil gelatinase lipocalin within 12 h of surgery. Similarly, the causal relationships between AKI and hard outcomes in the analyzed population were verified and quantified. The authors, for the first time, produce a repeatable coefficient (0.741) that may become a useful quality benchmark and could be applied to test future interventions aiming to reduce the burden of AKI on children’s clinical course.

## Main text

In this issue of *Critical Care*, Bojan and coauthors investigated the complex relationships between acute kidney injury (AKI), modeled as a latent variable, and several exposures, including hard outcomes, in a retrospective analysis of infants undergoing cardiopulmonary bypass surgery [1]. To do so, they applied structural equation modeling (SEM), which is a relatively novel way to apply statistical analysis to clinical medicine.

Clinical events are caused by a huge variety of combined variables and may generate a similar cascade of more or less predictable consequences. Attempts by researchers to force such clinical events into a mathematical model have often been frustrated or resulted in simplistic models: currently, the only way to generate a high level of evidence in order to show the effects of a treatment or the actual burden of a complex clinical condition (i.e., AKI) is to conduct randomized controlled studies or pragmatic prospective studies on a very large number of patients. Studies on pediatric critically ill patients are often retrospective, rarely randomized [2], and, in the vast majority of cases, conducted on small samples [3]. As alternatives, studies including the multi-faceted combination of several clinical factors and confounders are needed.

SEM, through complex modeling and calculations, is used to depict relationships among multiple simultaneously observed variables in order to provide a quantitative test of a theoretical model hypothesized by the researcher [4]. In other words, SEM may allow researchers to *verify* if it is correct that AKI negatively affects clinical outcomes and to *quantify* its effects. More specifically, SEM applies two major types of variables: latent variables and observed variables. Latent variables are variables that are not directly observable or measured (i.e., intelligence, productivity, multi-factorial syndromes) and, hence, are inferred from a set of observed variables that can actually be measured using tests, surveys, and so on [4].

In the article by Bojan and coworkers, several perioperative variables were available for analysis of 75 neonates and 125 infants with congenital heart diseases. This particularly severely ill cohort, which was rather homogeneous according to age, included patients requiring complex surgery and undergoing averagely long cardiopulmonary bypass and with a 20 % incidence of post-operative AKI. Interestingly, Bojan and coauthors modeled the latent variable AKI as a construct of a post-operative serum creatinine increase of 50 % over baseline, urine output <0.5 ml/kg/h, and urine creatinine-normalized neutrophil gelatinase lipocalin within 12 h of surgery. These three elements were extracted as the best performers in identifying the latent AKI factor during an exploratory analysis preceding SEM: interestingly, as for the purposes of the current analysis, they outperformed the Acute Kidney Injury Network classification, which was not included in the SEM analysis.

Recent published literature on pediatric cardiac surgery-associated AKI has already advocated the need for more extensive and timely examination of renal dysfunction, including the use of different criteria to AKI classification staging, such as fluid overload computation and biomarker application [5, 6]. The detection of early or subclinical forms of renal involvement [7, 8] in the pathophysiologic processes following bypass cardiac surgery directly and indirectly affect patients’ outcomes [9, 10] and SEM was likely able to detect this sequence of events. Bojan and coauthors also hypothesized that cardiopulmonary bypass would have a significantly greater role than low cardiac output on AKI development. It must be acknowledged that no direct measure of cardiac output was included in their study and its assessment is currently poorly applied in the context of pediatric cardiac surgery [11] and pediatric intensive care [12, 13]: it would be interesting to perform a similar analysis including direct cardiac output estimation.

What their study actually adds to the current literature, however, is the quantification of the association between “latent” AKI and hard outcomes (namely, duration of mechanical ventilation, length of intensive care unit stay, and in-hospital mortality): the authors provided the readers with a measurement of this association (a compound coefficient of 0.741). This value might be interpreted as a quality benchmark for comparison of the performance of other centers. Otherwise, it could be re-evaluated in future studies, including a larger number of subjects, a more complete set of AKI covariates (fluid overload amount, number or amount of nephrotoxic agents, measurement of advanced hemodynamic variables), or a different population (i.e., neonates with hypoplastic heart syndrome [14]). Finally, the impact of specific therapeutic or preventive interventions might be measured using SEM in order to ultimately verify if a reduction in renal dysfunction occurrence is actually possible and if the burden of AKI on patients’ outcomes may be ultimately limited.

## Conclusions

This study has added another small piece of knowledge to the pediatric cardiac AKI literature. Renal dysfunction has to be acknowledged as a complex and multifactorial syndrome that requires careful observation and accurate diagnosis, possibly not limited to classification staging but also implementing early biomarker analysis. Subclinical as well as established AKI significantly affects patients’ clinical courses and novel preventive or therapeutic interventions are urgently needed to finally improve outcomes.

## Abbreviations

AKI, acute kidney injury; SEM, structural equation modeling
